# Supplementary material for: Time-Resolved Proteomics of Germinating Spores of Bacillus cereus
Source: Int J Mol Sci. 2022 Nov 6;23(21):13614. doi: 10.3390/ijms232113614 (PMC9658361; doi:10.3390/ijms232113614)
Supplement: Supplementary file 1 [file ijms-23-13614-s001.zip › Time-resolved proteomics of germinating spores of Bacillus cereus_IJMS_supplementary figures.pdf]

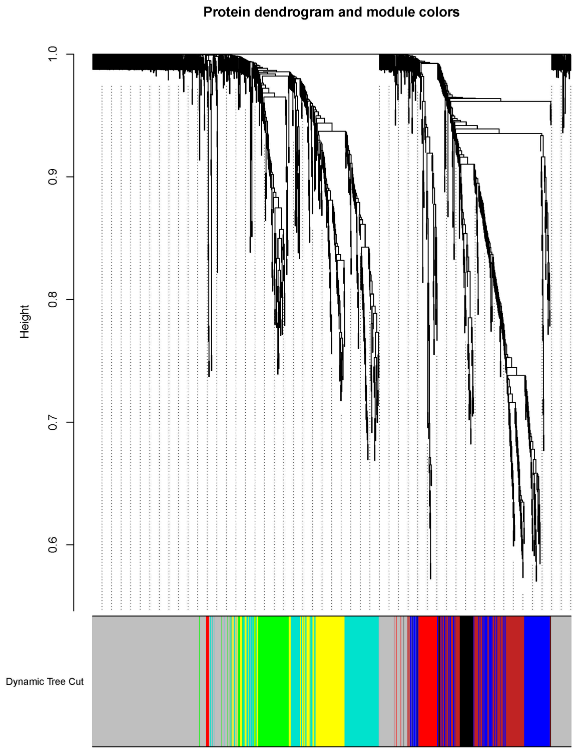

Figure S1. Co-expression network analysis protein dendrogram clustering. Based on topological overlap, protein dendrogram were clustered into 8 modules.

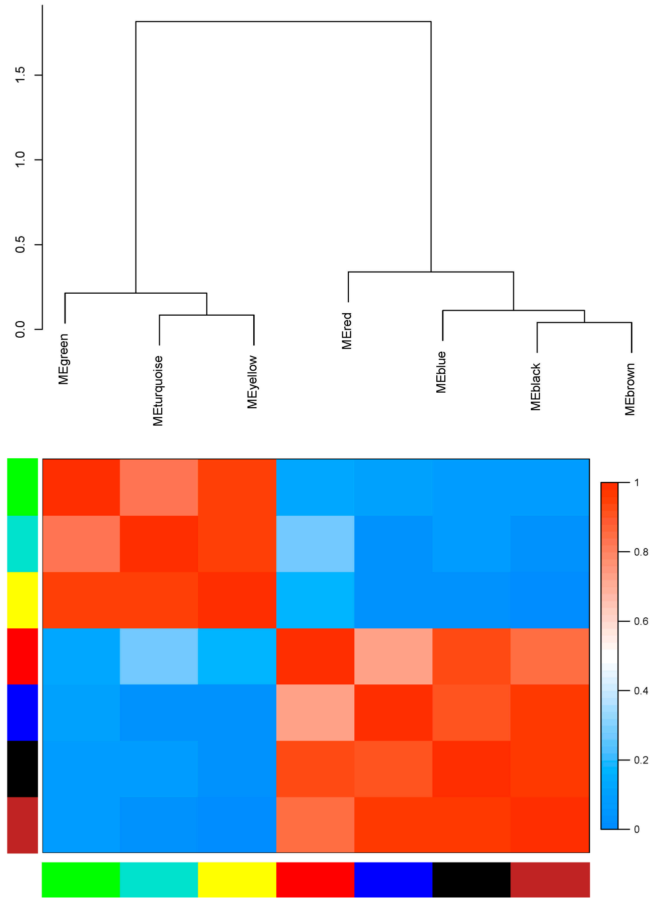

Figure S2. Eigenprotein correlation. The red color represents high correlation and blue for low correlation.

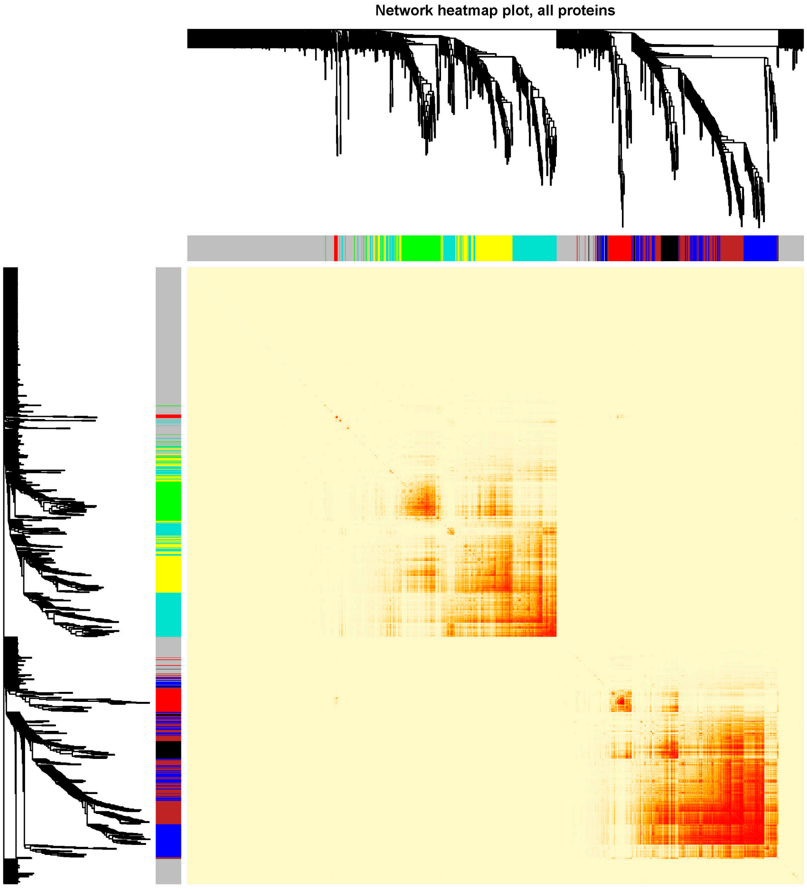

Figure S3. Topological overlap matrix (TOM) was visualized in a heatmap plot. Light color represents low overlap and red color represents higher overlap.

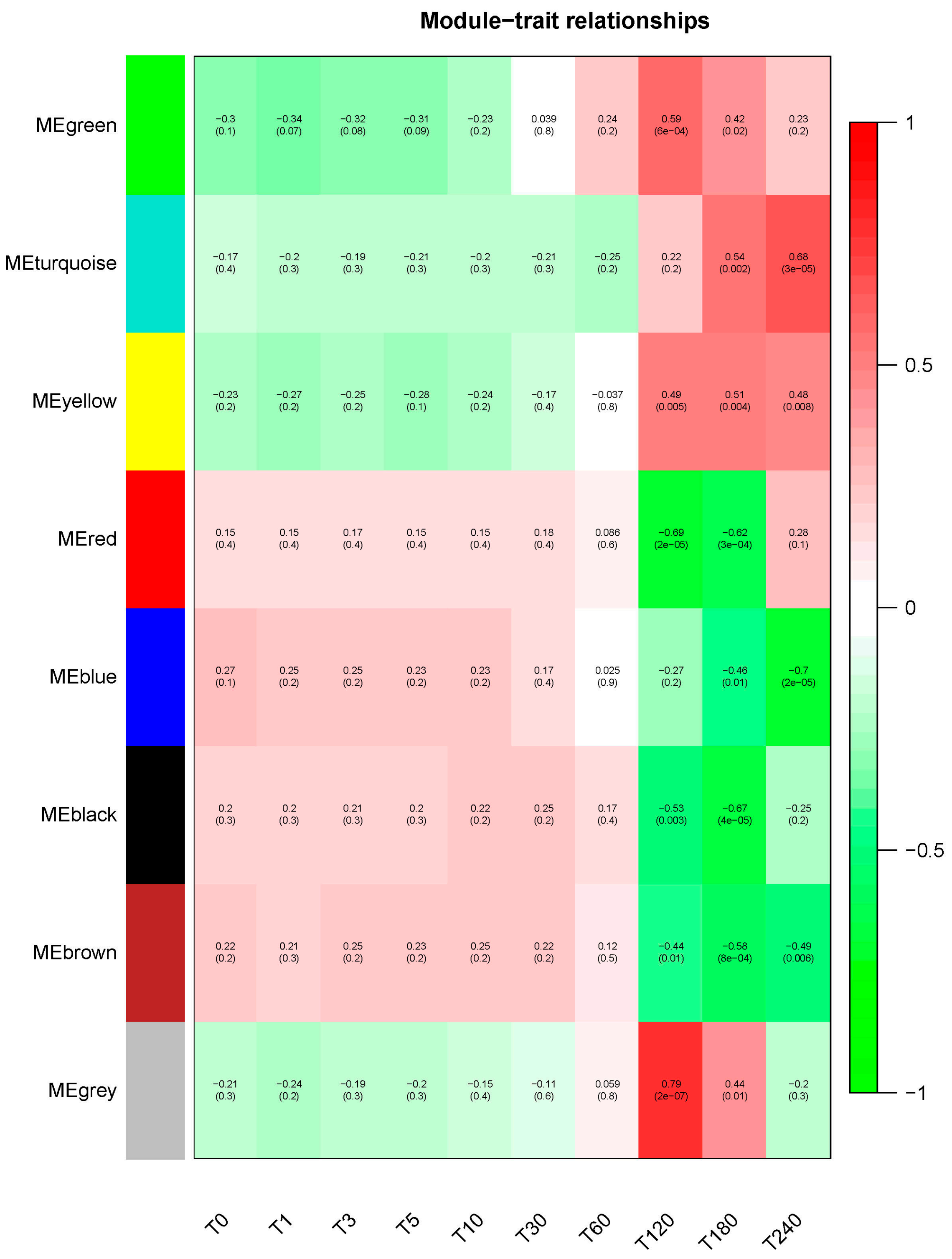

Figure S4. Co-expression network module-time correlation. Each cell showed the corresponding correlation (up) and *p*-value (down). Each cell was color coded based on correlation. The red color represents high correlation and blue for low correlation. The columns and rows indicate time points and module Eigenproteins respectively.
